# Huntington's disease accelerates epigenetic aging of human brain and disrupts DNA methylation levels

**DOI:** 10.18632/aging.101005

**Published:** 2016-07-27

**Authors:** Steve Horvath, Peter Langfelder, Seung Kwak, Jeff Aaronson, Jim Rosinski, Thomas F. Vogt, Marika Eszes, Richard L.M. Faull, Maurice A. Curtis, Henry J. Waldvogel, Oi-Wa Choi, Spencer Tung, Harry V. Vinters, Giovanni Coppola, X. William Yang

**Affiliations:** ^1^ Human Genetics, David Geffen School of Medicine, University of California Los Angeles, Los Angeles, CA 90095, USA; ^2^ Biostatistics, Fielding School of Public Health, University of California Los Angeles, Los Angeles, CA 90095, USA; ^3^ CHDI Management/CHDI Foundation, Princeton, NJ 08540, USA; ^4^ Department of Anatomy and Medical Imaging, Faculty of Medical and Health Science (FMHS), University of Auckland, Auckland, New Zealand; ^5^ Centre for Brain Research, Faculty of Medical and Health Science (FMHS), University of Auckland, Auckland, New Zealand; ^6^ Center for Neurobehavioral Genetics, Semel Institute for Neuroscience & Human Behavior, University of California, Los Angeles (UCLA), Los Angeles, CA 90095, USA; ^7^ Pathology and Laboratory Medicine, and Neurology, UCLA David Geffen School of Medicine, Los Angeles, CA 90095, USA; ^8^ Department of Psychiatry and Biobehavioral Sciences, David Geffen School of Medicine at UCLA, Los Angeles, CA 90095, USA; ^9^ UCLA Brain Research Institute, Los Angeles, CA 90095, USA

**Keywords:** Huntington's disease, epigenetic clock, DNA methylation, epigenetics, biomarker of aging, brain

## Abstract

Age of Huntington's disease (HD) motoric onset is strongly related to the number of CAG trinucleotide repeats in the *huntingtin* gene, suggesting that biological tissue age plays an important role in disease etiology. Recently, a DNA methylation based biomarker of tissue age has been advanced as an epigenetic aging clock. We sought to inquire if HD is associated with an accelerated epigenetic age. DNA methylation data was generated for 475 brain samples from various brain regions of 26 HD cases and 39 controls. Overall, brain regions from HD cases exhibit a significant epigenetic age acceleration effect (p=0.0012). A multivariate model analysis suggests that HD status increases biological age by 3.2 years. Accelerated epigenetic age can be observed in specific brain regions (frontal lobe, parietal lobe, and cingulate gyrus). After excluding controls, we observe a negative correlation (r=−0.41, p=5.5×10^−8^) between HD gene CAG repeat length and the epigenetic age of HD brain samples. Using correlation network analysis, we identify 11 co-methylation modules with a significant association with HD status across 3 broad cortical regions. In conclusion, HD is associated with an accelerated epigenetic age of specific brain regions and more broadly with substantial changes in brain methylation levels.

## INTRODUCTION

Huntington's disease (HD) is a dominantly inherited neurodegenerative disorder clinically characterized by progressive movement disorder, cognitive dysfunction, and psychiatric impairment [1]. HD is caused by a CAG trinucleotide repeat expansion resulting in an elongated polyglutamine stretch near the N-terminus of the huntingtin (HTT) protein [2]. HD patients have CAG repeat lengths greater than 36 on one of the *HTT* alleles. Although HD affects a number of brain regions such as the cortex, thalamus, and subthalamic nucleus, the striatum is the most severely affected region [3]. Large postmortem pathological series and neuroimaging studies suggest that CAG repeat length is highly correlated with caudate but not cortical atrophy [4-6]. The hallmark of HD neuropathology is massive degeneration of the striatal medium-sized spiny neurons (MSNs) and, to a lesser extent, the deep layer cortical pyramidal neurons [7]. HD neurodegeneration mainly affects the MSNs of the neostriatal nuclei, caudate nucleus and putamen, explaining the grave motor symptoms. Despite the specificity of neurodegeneration in HD, HTT is broadly present in cells throughout the brain [8].

HD is one of several polyglutamine disorders (including inherited ataxias, muscular dystrophy, and several forms of mental retardation [3]) that are caused by the expansion of unstable CAG trinucleotide repeats. The differential pathogenesis of polyglutamine disorders may be due to differences in polyglutamine protein context or functions because these disorders exhibit distinct patterns of neuronal loss and clinical manifestation despite nearly ubiquitous expression of these proteins, at least in the brain, and in the case of HTT the ubiquitous expression throughout the body and during development.

The age of onset of HD motor symptoms strongly correlates with the number of CAG trinucleotide repeats in *HTT* [9-11]. HD patients are usually clinically diagnosed in their 40s, but the age of onset can range from earlier than 10 for individuals with high repeat lengths to over 80 years for those with repeat lengths below 40. Overall, three non-mutually exclusive hypotheses could explain adult onset in HD: First, normal aging renders MSNs more vulnerable to mutant HTT toxicity [12]. Second, mutant HTT progressively produces cumulative defects over time. Third, mutant HTT toxicity accelerates the biological age of affected cells and tissues, which makes them vulnerable to dysfunction and cell death. We are not aware of any data or results that would support this third hypothesis. Irrespective of the validity of this “accelerated biological age hypothesis in HD”, there is little doubt that biological age plays an important role in HD. For example, the product of CAG repeat length and chronological age (“CAP score”) relates to clinical outcomes in HD according to recent longitudinal studies of HD patient cohorts [10]. Here, we address the challenge of directly testing whether HD is associated with accelerated aging in brain tissue by exploiting our DNA methylation based biomarker of tissue age, which is referred to as the epigenetic clock.

DNA methylation levels lend themselves to defining a biomarker of tissue age because chronological age has a profound effect on DNA methylation levels [13-17]. We recently developed an epigenetic measure of tissue age by combining the DNA methylation levels of 353 dinucleotide markers known as cytosine phosphate guanines or CpGs [18]. The weighted average of these 353 epigenetic markers gives rise to an estimate of tissue age (in units of years), which is referred to as “DNA methylation age” or as “epigenetic age”. This epigenetic clock method to estimate age appears to apply to any tissue or cell type that contains DNA (with the exception of sperm) including individual cell types (helper T cells, neurons, glial cells), complex tissues and organs (blood, brain, bone, breast, kidney, liver, lung [18-20]) and extending to prenatal brain samples [21]. The epigenetic clock method for estimating age is particularly attractive in the context of neuro-degenerative diseases for the following reasons. First, it applies to all brain regions, sorted brain cells [18-20], beginning with prenatal brain samples [21]. Second, recent findings suggest that the epigenetic clock captures aspects of the biological age of brain tissue, e.g. the epigenetic age of the frontal lobe relates to neuropathological variables and to Alzheimer's disease (AD) related cognitive functioning [22].

To explore changes in the brain methylome in HD individuals, we also carried out a systems biological analysis of DNA methylation levels. We constructed co-methylation modules and identified those that are associated with HD status in several brain regions.

## RESULTS

### Accuracy of the epigenetic clock in brain samples from HD patients and controls

**Figure 1:**
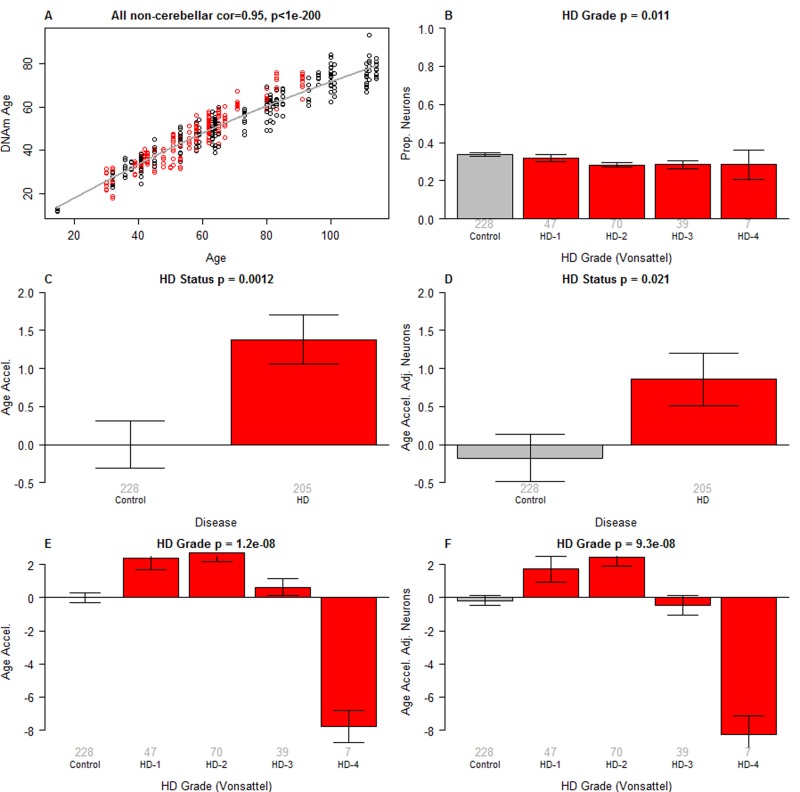
Epigenetic clock analysis of non-cerebellar brain regions (**A**) Scatter plot of (winsorized) DNAm age versus chronological age (x-axis). Red dots correspond to HD cases, black dots to non-HD samples. The curve corresponds to a spline regression line (2 degrees of freedom) through the non-HD samples. Epigenetic age acceleration was defined as the vertical distance of each sample from the spline regression line. (**B**) HD Vonsattel grade vs the proportion of neurons (y-axis). The proportion of neurons was estimated based on DNA methylation data using the CETS method [23]. (**C**,**D**) HD status versus (**C**) epigenetic age acceleration, D) an intrinsic measure of epigenetic age acceleration that adjusts for the proportion of neurons. (**E**,**F**) HD Vonsattel grade versus (**E**) age acceleration and (**F**) an intrinsic measure of epigenetic age acceleration that adjusts for the proportion of neurons. All bar plots show the mean value (y-axis) and one standard error and report the results from a non-parametric group comparison test (Kruskal Wallis). The “winsorized” the DNAm age estimates changed the values of four putative outliers as described in Supplementary Figure 1.

We collected 475 brain samples from multiple brain regions of 65 individuals (26 HD, 18 Alzheimer's disease, and 21 controls) and profiled the samples using the Illumina 450k platform. An overview of our data set is presented in Table 1. Individual level data such as postmortem interval can be found in Supplementary Table 1. Epigenetic age (referred to as DNAm age) was calculated as described in [18]. As expected, DNAm age has a strong linear relationship with chronological age in brain tissue samples (r=0.94, Supplementary Figure 1A). However, 4 samples deviate strongly from the linear trend. To err on the side of caution, we “winsorized” the DNAm age estimates of these 4 putative outliers by replacing them with the second most extreme age estimate from the same individual (based on the remaining non-cerebellar brain regions). Winsorisation effectively limits the adverse effects of severe outliers in the DNAm age estimate. We did not use DNAm age estimates from the cerebellum in this winsorization approach because the cerebellum ages more slowly than other brain regions [20]. After the winsorization, the correlation between DNAm age and chronological age increased slightly (from r=0.94 to r=0.95, Figure 1A).

**Table 1:** Overview of the brain methylation data set

	Disease Status		
	Huntington's	Alzheimer's	Control
**Brain samples (n)**	215	125	135
**Frontal lobe (n)**	50	21	32
**Occipital lobe (n)**	31	24	20
**Parietal lobe (n)**	62	0	35
**Temporal lobe (n)**	8	23	6
**Caudate nucleus (n)**	17	0	12
**Cerebellum (n)**	10	23	9
**Cingulate gyrus (n)**	21	0	12
**Hippocampus (n)**	8	18	7
**Midbrain (n)**	8	16	1
**No. of individuals**	26	18	21
**No. of women**	10	13	6
**Mean Age (range)**	56.1 (30, 91)	84.6 (58, 114)	59.1 (15, 93)
**Mean Postmortem interval**	14.8 (3.5, 46)	20.5 (21, 52)	16.4 (6.0, 36)

To formally measure epigenetic age acceleration effects, we constructed a regression model of DNAm age on chronological age in non-HD samples (grey line in Figure 1A). We then defined age acceleration for each sample (HD or non-HD) as the corresponding residual resulting from the regression model. Thus, positive age acceleration means the (methylation state of the) sample appears to be older than would be expected from non-HD samples. We find that HD is significantly associated with epigenetic age acceleration (Figure 1C), and that this finding holds even when one uses the un-winsorized version of DNAm age (Supplementary Figure 1B). We also defined an “intrinsic” measure of age acceleration as the residual that results by regressing DNAm age on both chronological age and the proportion of neurons which was estimated using the CETS method [23]. The resulting cell-intrinsic measure of age acceleration, which is not confounded by the abundance of neurons, is again associated with HD status (Figure 1D). We find that epigenetic age acceleration relates significantly to Vonsattel grade (VS grade), a semi-quantitative (0-4) measure of neuro-pathologic abnormalities of post-mortem HD brains based on macroscopic and microscopic criteria [24]. VS grade 1 and 2 samples exhibit the highest positive age acceleration whereas VS grade 4 samples exhibit *negative* epigenetic age acceleration (Figure 1E) which persists even after controlling for the proportion of neurons/glia (Figure 1F). This unexpected negative age acceleration in VS grade 4 samples, which can also be observed in specific brain regions (Supplementary Figure 2), may be due to one of the following explanations. First, it could be a false positive that reflects the low sample size (n=7) of grade 4 samples.

**Figure 2:**
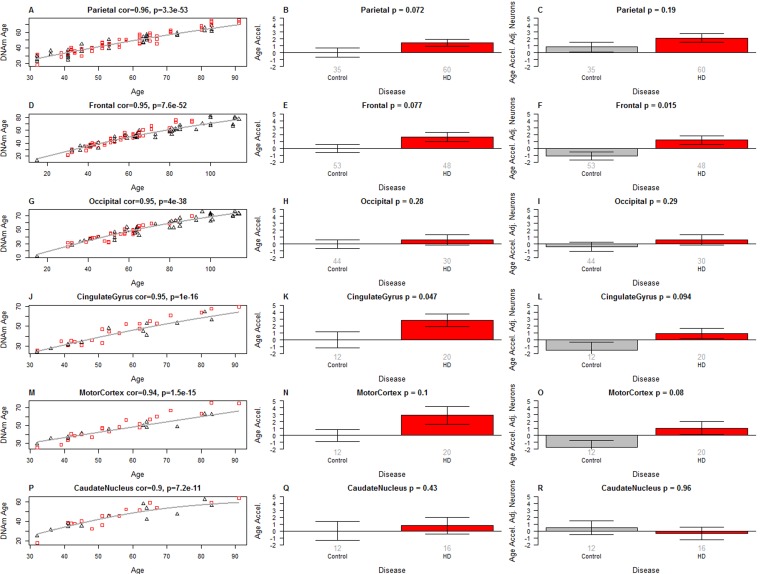
Epigenetic age acceleration in specific brain regions Rows correspond to different brain regions. The first column (**A,D,G,J,M,P**) depicts DNAm age (y-axis) versus chronological age (x-axis) in different brain regions. The grey line corresponds to a spline regression model (based on 2 degrees of freedom) through non-HD samples. Epigenetic age acceleration was defined as the vertical distance of each sample from the spline regression line. The bar plots in the second column (**B,E,H,K,N,Q**) show the relationship between epigenetic age acceleration (y-axis) and HD status. The bar plots in the third column (**C,F,I,L,O,R**) involve the intrinsic measure of age acceleration that adjusts for the proportion of neurons. The rows correspond to samples from the parietal lobe, frontal lobe, occipital lobe, cingulate gyrus, motor cortex, and caudate nucleus. Each bar plot depicts the mean value and one standard error and reports a non-parametric group comparison test p-value (Kruskal Wallis Test). HD grade 4 samples were removed from this analysis.

However, we think this explanation is unlikely since one already observes a diminished epigenetic age acceleration in grade 3 samples and because we find a similar negative relationship of epigenetic age acceleration with CAG repeat length (as described below). Second, it might reflect the severe loss of neurons even though moderate changes in cell composition do not seem to affect the estimate of DNAm age [18, 20]. However, we observe the same effect when using our cell intrinsic measure of age acceleration that adjusts for the proportion of neurons (Figure 1F). Further, only a marginally significant association between the proportion of neurons and VS grade can be observed in the brain regions of our study (p=0.011, Figure 1B). We next studied epigenetic age acceleration in individual brain regions. After removing grade 4 samples, we find that HD has a suggestive association with epigenetic age acceleration in the parietal lobe (p=0.072, Figure 2B), frontal lobe (p=0.077, Figure 2F), and cingulate gyrus (p=0.047, Figure 2K). No significant associations could be observed in the occipital lobe (Figure 2H,I). Comparisons in other brain regions, including the caudate nucleus (Figure 2Q,R), were inconclusive, possibly due to HD disease stage (the striatum is more affected than the cortex and may thus be equivalent to HD stage 3 or 4) or due to the low group sizes (group sizes are shown under each bar in the bar plot panels in Figure 2).

### Regression analysis that adjusts for possible confounders

We next asked whether the observed epigenetic age acceleration could be due to confounding by known or unknown confounders. To answer this question, we studied age acceleration using three different multivariate linear regression models that include known and inferred confounders (Table 2). The first model regressed DNAm age on HD status, chronological age, sex, brain bank, and brain region. We find that HD status remains highly significantly associated with DNAm age (p=6.7×10^−5^) even after adjusting for these known confounders. In the second linear model, which contains the estimated proportion of neurons as covariate, HD status remains highly significantly associated with DNAm age (p=0.00070). The third model is similar to the first but also adjusts for the first five principal components (PCs) estimated from the DNA methylation data. These PCs are likely to reflect unobserved con-founders (technical variation, changes in cell composition) and so can be viewed as inferred confounders. Although including 5 PCs in a multivariate model may be overly conservative, HD status remains marginally significantly associated with DNAm age (p=0.065). Overall, the multivariate model analysis strongly suggests that the epigenetic age acceleration effects observed in HD are not due to confounding effects. The multivariate models allow us to estimate the increase in biological age due to HD status. HD status increases the biological age by 3.2 years according to model 1 or by 2.7 years according to model 2 (caption of Table 2).

**Table 2:** Linear model that regresses DNAm age on HD status and other covariates

		Model 1		Model 2		Model 3	
Covariate	Contrast	Coef (SE)	P-value	Coef (SE)	P-value	Coef (SE)	P-value
Huntington		2.06 (0.517)	6.7×10^−5^	1.704 (0.503)	0.00070	0.9 (0.486)	0.065
Age		0.646 (0.012)	<2×10^−16^	0.64 (0.011)	<2×10^−16^	0.632 (0.011)	<2×10^−16^
Sex	Female vs Male	−0.981 (0.49)	0.046	−0.84 (0.474)	0.077	0.611 (2.637)	0.817
Brain Bank	UCLA vs NewZealand	−0.093 (1.224)	0.94	1.049 (1.198)	0.382	1.32 (1.139)	0.247
Tissue	Caudate Nucleus vs Frontal	−1.237 (1.266)	0.33	−3.412 (1.278)	0.008	−3.239 (1.201)	0.007
	Cingulate Gyrus vs Frontal	−1.631 (1.224)	0.18	−1.961 (1.183)	0.098	−0.729 (1.119)	0.52
	CRBM vs Frontal	−5.353 (1.121)	1.8×10^−6^	−3.854 (1.113)	0.001	16.194 (10.299)	0.12
	Hippocampus vs Frontal	1.327 (1.191)	0.27	−0.077 (1.175)	0.95	1.08 (1.131)	0.34
	Midbrain vs Frontal	−1.115 (1.274)	0.38	−4.12 (1.334)	0.002	−1.327 (1.331)	0.32
	Motor Cortex vs Frontal	1.539 (1.224)	0.21	1.699 (1.182)	0.151	1.64 (1.112)	0.14
	Occipital vs Frontal	−2.886 (1.115)	0.01	−1.704 (1.096)	0.121	−2.218 (1.037)	0.033
	Parietal vs Frontal	0.835 (1.065)	0.43	1.781 (1.042)	0.088	1.382 (0.982)	0.16
	Sensory Cortex vs Frontal	−0.173 (1.224)	0.89	0.079 (1.183)	0.95	0.179 (1.116)	0.87
	Temporal vs Frontal	0.191 (1.156)	0.87	0.228 (1.116)	0.84	0.621 (1.053)	0.56
	Visual Cortex vs Frontal	0.4 (1.233)	0.75	2.178 (1.23)	0.077	0.408 (1.192)	0.73
Prop. Neurons				−13.966 (2.395)	5.5×10^−9^		
PC1						862.505 (313.408)	0.006
PC2						147.452 (64.232)	0.022
PC3						−36.821 (13.872)	0.008
PC4						59.519 (11.864)	5.3×10^−7^
PC5						8.251 (26.496)	0.76

Coefficients, standard error, and corresponding p-values for three multivariate models.

According to model 1, the age acceleration due to HD status amounts to 3.3 years (=2.159/0.646).

Model 2 is similar to model 1 but includes the (estimated) proportion of neurons as covariate. Model 3 is similar to model 1 but includes principal components. Since the analysis ignores the dependency of observations (due to multiple brain regions coming from the same individual), the p-values should only be interested as descriptive measures (as opposed to inferential measures). The multivariate models allow us to estimate the increase in biological age due to HD status. HD status is associated with an increase of 3.2 years (=2.06/0.646) according to model 1, an increase of 2.7 years (=1.704/0.64) according to model 2, and am increase of 1.4 years according to model 3.

### CAG-repeat length versus epigenetic age acceleration

**Figure 3:**
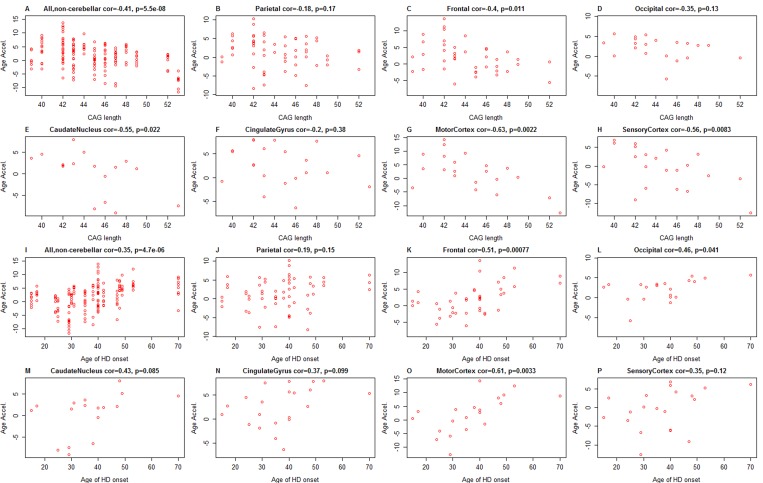
CAG length and age of HD onset versus epigenetic age acceleration in HD patients Results for CAG length and for age of onset can be found in the first two rows and the last two rows, respectively. (**A**-**H**) CAG length (x-axis) versus epigenetic age acceleration in (**A**) all non-cerebellar samples, (**B**) parietal lobe, (**C**) frontal lobe, (**D**) occipital lobe, (**E**) caudate nucleus, (**F**) cingulate gyrus, (**G**) motor cortex, (**H**) sensory cortex. (**I**-**P**) Age of HD motoric onset (x-axis) versus epigenetic age acceleration in (**I**) all non-cerebellar samples, (**J**) parietal lobe, (**K**) frontal lobe, (**L**) occipital lobe, (**M**) caudate nucleus, (N) cingulate gyrus, (**O**) motor cortex, (**P**) sensory cortex.

The graded impact of CAG length on HD age of onset and disease manifestation leads to the “polyglutamine trigger” hypothesis, which suggests that polyglutamine expansion in the context of endogenous HTT protein leads to subtle but repeat-length-dependent graded molecular changes in affected cells that act in a dominant fashion to trigger the disease [25]. The search of CAG-repeat-length dependent, continuous molecular changes have implicated altered energetics [26], gene expression, and epigenetic changes [27-30]. After removing controls, we find a significant negative correlation between CAG length and epigenetic age acceleration of HD brain samples (r=−0.41, p=5.5×10^−8^, Figure 3A) and in specific brain regions from HD cases (Figure 3B-H). This negative correlation probably relates to the finding that VS grade 4 samples exhibit negative age acceleration because a) the seven grade 4 samples also exhibited the highest CAG length (of 53 trinucleotide replicates), and b) VS grade is strongly correlated with CAG length in our HD cases (r=0.75, p=9.0×10^−5^, Supplementary Figure 3A).

For a subset of 21 HD subjects, we also had information on the age of HD motoric onset. We found a significant positive correlation between the age of HD motoric onset and epigenetic age acceleration (Figure 3I-P). The marginal associations between age acceleration and the clinical parameters (age of onset, CAG length, and HD grade) are congruent with the pairwise correlations between the clinical parameters in the 21 HD subjects (Supplementary Figure 3): CAG length has a strong positive correlation with HD grade (r=0.75) and a strong negative correlation with age of onset (r=−0.55, p=0.0098, Supplementary Figure 3B). Age of onset was highly correlated with chronological age at death in our data set (r=0.78, p=3.5×10^−5^). No significant correlation could be observed between HD grade and age of onset (Supplementary Figure 3C).

In contrast to our findings of epigenetic age acceleration in brains of HD cases, we find no difference in epigenetic age acceleration between Alzheimer's disease brains and controls (Supplementary Figure 4), which might reflect the low sample size as discussed below. We could not find a significant age acceleration effect due to HD in several brain regions (Supplementary Figure 5), which might reflect the low sample sizes.

### Epigenome-wide association study (EWAS)

In a secondary analysis, we related HD status to individual epigenetic markers (CpGs). Here we focused on 327k CpGs (out of over 485k) with highest variance across the samples (Methods).

**Figure 4:**
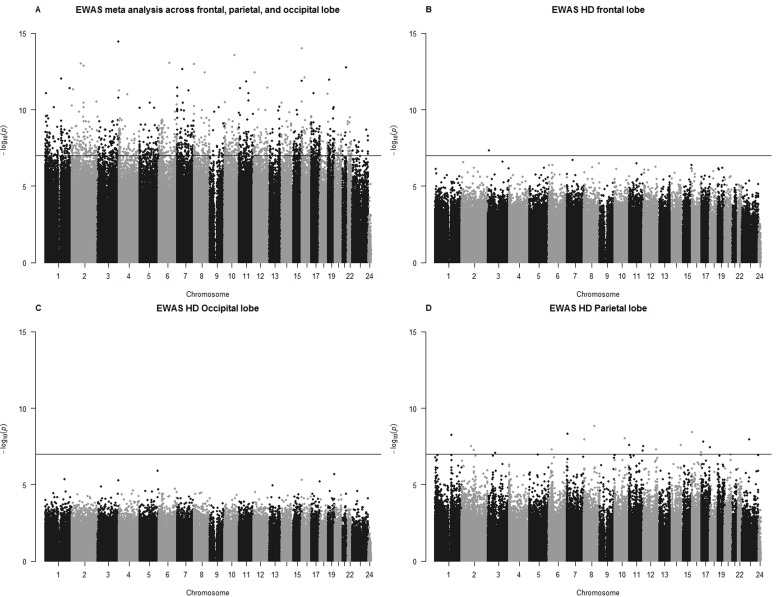
Manhattan plots for EWAS results (**A**) The y-axis shows log (base 10) transformed p-values resulting from a meta analysis across 3 lobes (frontal, parietal, and occipital lobe). Meta analysis p-value resulted from the *limma* R function that also included the batch as covariate. EWAS results (Kruskal Wallis test) for individual lobes can be found in (**B**) frontal lobe, (**C**) occipital lobe, (**D**) parietal lobe. The horizontal line corresponds to a Bonferroni corrected significance level of p=0.05/500000. The statistical analysis ignored the dependence between observations arising from the fact that multiple samples were collected from the same individual. Therefore, the p-values should be considered as descriptive (rather than inferential) measures.

Since sex and age has profound effects on DNA methylation levels (which are largely preserved across brain regions Supplementary Figure 6), we adjusted the DNA methylation levels for age and sex by forming residuals. Further, we restricted the analysis to samples from post mortem lobes for which we had sufficient sample sizes (Table 1) namely the frontal lobe (Figure 4B), occipital lobe (Figure 4C), and parietal lobe (Figure 4D). The association between HD and age-adjusted methylation levels is strongly preserved across the lobes (Supplementary Figure 7). After combining the EWAS results from each of the 3 lobes using meta analysis, we found that 1467 CpGs are significantly associated with HD at a Bonferroni corrected significance level of 1×10^−7^ =0.05/500000 (Figure 4A). The 16 most significant (p <1.2×10^−12^) HD related CpGs are presented in Table 3 and in Supplementary Figure 8. The meta-analysis p-values need be interpreted as descriptive (hypothesis promoting) rather than inferential measure for the following reasons. First, the meta analysis did not adjust for the fact that multiple samples were collected from each individual. Second, the distribution of EWAS p-values exhibit high inflation factors (lambda=7.3 for the meta analysis, 3.5 for the frontal lobe, 3.0 parietal lobe, 2.2 for the occipital lobe, Supplementary Figure 9). Detailed results for all CpGs can be found in Supplementary File 11.

**Table 3:** The most significant CpGs from our EWAS of HD status across three brain regions

CpG name	Gene	Chrom.	Z statistic meta	p meta analysis	p Frontal	p Occipital	p Parietal
cg01524723		3	7.87	3.6E-15	7.2E-07	5.3E-06	3.5E-05
cg10112599	TMEM8A	16	7.74	9.9E-15	1.1E-06	4.8E-06	6.9E-05
cg11540707	IDE	10	7.61	2.8E-14	2.8E-05	1.7E-03	9.5E-09
cg22897634	GRIK2	6	7.45	9.2E-14	1.8E-06	5.7E-05	4.4E-05
cg05482066		8	7.43	1.1E-13	1.3E-06	6.2E-04	6.6E-06
cg27250180		21	7.36	1.8E-13	8.0E-06	4.2E-05	2.8E-05
cg14593290	DDC	7	7.33	2.4E-13	1.9E-07	3.7E-04	1.2E-04
cg00249621	TSPYL5	8	7.27	3.7E-13	1.3E-04	1.0E-02	1.6E-09
cg00160777	CHP2	16	7.16	8.1E-13	5.0E-06	8.4E-03	4.8E-07
cg14937409	KRI1	19	7.11	1.2E-12	8.3E-07	2.8E-04	2.2E-04
cg04195855	LRRK1	15	7.09	1.3E-12	7.0E-07	1.9E-03	4.1E-05
cg08291433		11	7.08	1.5E-12	4.4E-06	9.9E-04	1.8E-05
cg21535199	LCE1F	1	−7.14	9.4E-13	4.5E-05	2.3E-02	5.8E-09
cg08718119	LOC642846	12	−7.27	3.7E-13	8.4E-07	9.8E-03	1.0E-06
cg14227325	RGPD8	2	−7.4	1.3E-13	3.0E-05	1.9E-03	5.6E-08
cg17863923	RGPD1	2	−7.45	9.7E-14	1.7E-04	3.7E-04	3.2E-08

The second column reports the gene symbol of a neighboring gene. The CpGs were selected according to the meta analysis p-value (5^th^ column) across the 3 regions (frontal, occipital, and parietal lobe). The meta analysis Z statistic (4^rd^ column) is positive/negative for CpGs that are hyper/hypo methylated in HD compared to non-HD samples. “p Frontal” denotes the Kruskal Wallis p value for disease status in the frontal lobe samples. Bar plots can be found in Supplementary Figure 9.

### WGCNA reveals HD-dependent co-methylation modules

In light of the low sample size we conducted weighted correlation network analysis (WGCNA) [31-34], which is a systems biological analysis method that has been successfully applied to DNA methylation data, e.g. to study aging effects [35]. WGCNA constructs modules of co-methylated CpGs and identifies modules (as opposed to individual CpGs) that correlate with HD status. Among other advantages, this circumvents the problem of multiple comparisons (485k CpGs on the Illumina Infinium 450K array). We applied WGCNA to the same sex and age adjusted methylation data that were used in our EWAS. We again focused on samples from three lobes (frontal, occipital, parietal) for which we had sufficient sample sizes. To prevent between-lobe differences in methylation from confounding the module analysis, we employed a consensus network [36] analysis across the three lobes that essentially conditions out between-lobe differences. The analysis identified 54 co-methylation modules; by construction, these modules contain CpGs co-methylated in each of the three lobes. In this manner, network analysis reduced hundreds of thousands of variables across the 3 lobes to a relatively small number (n=54) of modules. Since the methylation profiles of probes in each module are strongly correlated in each of the 3 lobes, it is useful to summarize each module using a single representative profile. Toward this end, we defined the module representative as the first singular vector resulting from the singular value decomposition of the scaled methylation levels. We refer to this representative methylation profile, which can be interpreted as the weighted average of the CpGs inside a module, as the eigenvector (also known as eigengene or eigenprofile).

**Figure 5:**
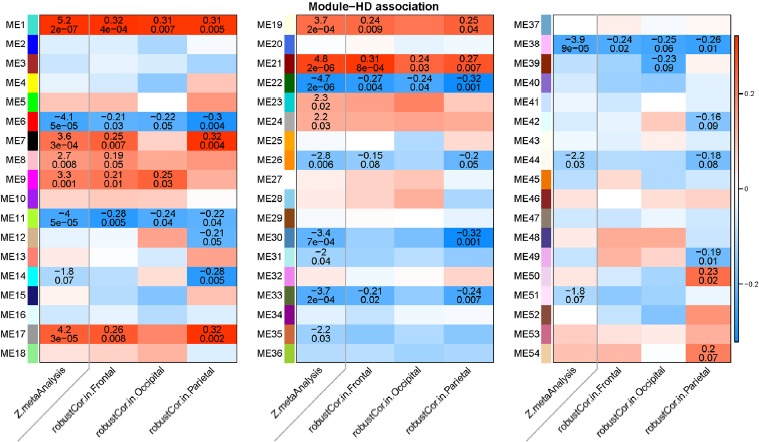
Heat map of correlations between modules and HD status in different lobes The rows correspond to modules found in a consensus module analysis across three lobes (frontal lobe, occipital lobe, parietal lobe). Each module (eigenvector) was correlated to HD status and to the proportion of neurons in the respective brain regions using a robust correlation test (biweight midcorrelation). Columns 2-4 in each panel report the robust correlation coefficients and the corresponding p-value (underneath the correlation coefficient) in the frontal, occipital, and parietal lobe, respectively. Each cell is color-coded according to the sign and strength of the correlation coefficient as shows in the color legend at the right hand side. Stouffer's meta analysis method was used to combine the three robust correlation test statistics across the three lobes. The first column of each panel presents a meta analysis Z statistic for HD status (Stouffer's method applied to the results from the 3 lobes) and corresponding p-value. The remaining columns present analogous results for the estimated proportion of neurons.

To identify modules related to HD status, we correlated the 54 module eigenvectors with HD status in the 3 lobes (Figure 5). We then used a meta-analysis of the eigenvector-HD correlations to quantify the overall relationship between a consensus module and HD status across all 3 lobes. Eleven modules passed a Bonferroni corrected meta-analysis significance threshold of p=0.05/54= 9.3×10^−4^ that adjusts for the number of modules (n=54). Five of these modules are hyper-methylated in HD: module 1 (meta-analysis p=2×10^−7^), module 21 (p=2×10^−6^), module 17 (p=3×10^−5^), module 19 (p=2×10^−4^), and module 7 (p=3×10^−4^). Six modules are hypo-methylated in HD (module 22 p=2×10^−6^, module 6 p=5×10^−5^, module 11 p=5×10^−5^, module 38 p=9×10^−5^, module 33 p=2×10^−4^, module 30 p=7×10^−4^). The network analyses provide several layers of information. First, the strength and significance of associations between modules and HD status are strongest in the parietal lobe, followed by the frontal lobe and then the occipital lobe. Second, the meta-analysis significance Z statistics allow us to rank modules by their overall association with HD status. Module 1 exhibits the strongest positive association whereas module 22 the strongest negative association with HD status (first column in the 3 heat maps of Figure 5). We also related the module eigengenes to the age of motor onset but found only suggestive associations that were not significant after adjusting for multiple comparisons (Supplementary Figure 10).

WGCNA provides a continuous (“fuzzy”) measure of module membership (MM) for all CpGs with respect to each of the modules. The module membership measures how similar the methylation profile of a CpG is to the eigenvector of the co-methylation module. CpGs whose profiles are highly similar to the eigenvector can be identified as intramodular hub nodes[33]; such hubs are often useful for implicating relevant biological pathways and prioritizing genes for functional studies[37]. The module membership measures of all CpGs can be found on our webpage HDinHD[38] (www.HDinHD.org). The module membership values of intramodular hubs can be found in Supplementary File 12.

### Enrichment analysis using the software tool HDinHD

We used a functional enrichment tool known as HDinHD[38] (www.HDinHD.org) to relate co-methylation modules to existing gene sets, either published or generated by other HDinHD users. We adapted the gene enrichment analysis to the special case of DNA methylation data as described in Methods. The most significant results from a hypergeometric test can be found in Table 4. Methylation module M1, which has the strongest positive association with HD status, is highly enriched with genes involved in sensory perception of chemical stimulus (p=6.2×10^−17^) and olfactory receptor activity (p=9.5×10^−16^). Interestingly, our methylation module 1 overlaps with a transcriptional module (also labelled module 1 in HDinHD) that has been found in several *co-expression* network analyses of transcriptomic data sets. In particular, it overlaps significantly (p=1.1×10^−45^) with a co-expression module (labelled M.1) that was found in striatal brain expression data from a mouse model of HD [39]. To be clear, our module 1 is distinct from the co-expression module M.1 but the two modules share a significant number of genes in common. Further, co-methylation module 1 overlaps with a striatal coexpression module also labelled M.1 (p=9.4×10^−24^) that was found in a consensus WGCNA across 3 mouse data sets. Further, it overlaps significantly with a cortical co-expression module labelled M.1 (p=1.9×10^−11^) which was found in a consensus network analysis across three developmental time points from an allelic series of HD mouse models [38]. Genes inside the striatal co-expression module (M.1) have a positive correlation with CAG length in the allelic series [38]. Further, it overlaps significantly with a human co-expression module found in the prefrontal cortex (also labelled M.1=1.1×10^−17^) and the visual cortex (p=2.4×10^−11^). Co-methylation module 1 is also enriched with genes that play a role in olfactory receptor activity and the detection of a chemical stimulus.

**Table 4:** Co-methylation modules that are enriched with gene lists from HDinHD

Module	Gene Set Identifier	Description	Source	p-value
1	WGCNA.HD.013.01	MODULE1 (co-expression)	WGCNA of mouse HD data from Giles 2012, Q150 striatum, adjusted for age	1.1×10^−45^
1	WGCNA.HD.010.01	MODULE1	WGCNA of mouse HD dataConsensus WGCNA across mouse R6/2, Q150, Allelic Series striatum	9.4×10^−24^
1	WGCNA.HD.019.01	MODULE1	WGCNA of mouse HD Consensus WGCNA of 2-, 6-, 10-month Allelic Series cortex	1.9×10^−11^
1	WGCNA.HD.004.01	MODULE1	WGCNA of human HD data: Harvard Brain Tissue Resource - Prefrontal Cortex	1.1×10^−17^
1	WGCNA.HD.005.01	MODULE1	WGCNA of human HD data: Harvard Brain Tissue Resource - Visual Cortex	2.4×10^−11^
1	GO:0050907	Detection of chemical stimulus involved in sensory perception	GO.BP	6.2×10^−17^
1	GO:0004984	olfactory receptor activity	GO.MF	9.5×10^−16^
6	JAM:002734	Annotated genes bound by RNA polymerase II	Table_S2 from Lee 2006	4.6×10^−56^
6	GO:0031981	nuclear lumen	GO.CC	1.4×10^−40^
6	GO:0090304	nucleic acid metabolic process	GO.BP	5.1×10^−37^
6	GO:0016070	RNA metabolic process	GO.BP	3.1×10^−33^
11	JAM:002734	Annotated genes bound by RNA polymerase II	Table_S2 from Lee 2006	7.8×10^−13^

Two HD related co-methylation modules (modules 6 and 11) are highly enriched in genes that are bound by RNA polymerase II (using a gene list from [40]).

### Relationship to prior work

Several articles point to an epigenetic modulation of HD pathophysiology [30], in the form of HDAC reduction [41] and/or epigenetic signatures [42, 43]. Our experimental analysis is focused on DNA methylation levels. Recent publications looked at methylation levels of *selected* genes in HD patients [44] and analyzed cortical samples from 7 HD patients and 6 controls [45]. Previous work has demonstrated that post-translational modifications of histone proteins are significantly altered in HD cellular and animal models as well as HD patients (reviewed in [46]). For example, H3K4me3, a marker of active gene expression [47], is reduced at promoters of selective downregulated genes in cortical and striatal regions in both R6/2 Htt model mice and HD patients [42]. Furthermore, studies have shown a potential therapeutic role for histone deacetylase (HDAC) inhibitors in numerous HD rodent and cell models (reviewed in [46]). DNA (de)methylation in HD has been investigated in transgenic models [48].

Modified bisulfite sequencing with single base pair resolution was employed to measure DNA methylation in the STHdh cellular model of HD [65]. The results from this study demonstrated that there was a bias towards hypomethylation associated with CpG-poor regions in the mHtt expressing STHdh111/111 compared to control STHdh7/7 cells.

Other DNA modifications may be relevant to HD pathology: global levels of 5hmC were reduced in the striatum and cortex of presymptomatic YAC128 mice [49] and 7-methylguanine was found to be reduced in the motor cortex from HD cases [50].

## DISCUSSION

To our knowledge this is the first study to demonstrate that HD is associated with epigenetic age acceleration in specific brain regions, namely frontal lobe, cingulate gyrus and the parietal lobe. Although the positive age acceleration effects that we observed could be the result of cell type abundance differences between HD and control samples, there are several reasons that make this unlikely. First, our intrinsic measure of age acceleration that adjusts for the abundance of neurons also reveals an accelerated aging effect. Second, epigenetic age acceleration can be observed in brain regions that are relatively unaffected by the disease (e.g. the parietal lobe Figure 2B). Third, our multivariate analysis suggests that the age acceleration effect is independent of the proportion of neurons and unobserved confounders. Finally, the epigenetic age *acceleration* in Vonsattel (VS) grades 1 and 2 and to a lesser extent in grade 3 cannot reflect the loss of neurons because grade 4 samples, which are associated with the most severe loss of medium spiny neurons, appear to exhibit *negative* epigenetic age acceleration (Figure 1E,F, Supplementary Figure 2). The negative age acceleration in VS grade 4 is unexpected and could be a false positive reflecting the very small sample size.

Our study contributes to an increasing body of evidence suggesting that epigenetic age acceleration is associated with neurodegenerative disorders [22, 51, 52]. Future research will be needed to evaluate to what extent increased epigenetic age acceleration is specific to HD. Using our own relatively small data set (Table 1), we find no difference between Alzheimer's disease brains and controls when it comes to epigenetic age acceleration (Supplementary Figure 4). However, we recently analyzed a large (n=700) number of prefrontal cortex samples from AD cases and controls to show that epigenetic age acceleration has significant correlations with neuropathologic variables and measures of cognitive functioning [22]. We also found evidence that epigenetic age is increased in brain samples from Down syndrome [51] and HIV+ individuals [53].

A question our study left unanswered is whether the aging acceleration in HD is specific to the methylation-based biomarker of age or whether it could be observed using other biomarkers of aging. Until recently few suitable biomarkers of tissue age have been available, making it challenging to directly test whether HD is associated with accelerated aging in brain tissue. Leukocyte telomere length could be a promising biomarker since telomere shortening is related to premature senescence and could be a marker of early cell death in neurodegenerative disorders. Indeed, recent evidence suggests that leukocyte telomeres are shortened in HD and several neurodegenerative disorders [54]. However, it remains to be seen to what extent leukocytes lend themselves as “surrogate” tissue for brain when it comes to assessing aging. Telomere length is probably not a suitable marker to directly measure the age of brain tissue because a) terminally differentiated neurons do not replicate and b) telomere measurements of brain tissue are inherently variable due to the cellular complexity within the sample [55].

A key advance of our study in the polyglutamine disease field is to apply epigenome-wide DNA methylation data from multiple brain regions of HD individuals and controls to identify HD related co-methylation networks. Our systems biological analysis identified 11 co-methylation modules that are strongly associated with HD status in several lobes. Interestingly, the most significant co-methylation module overlaps with a co-expression module found in transcriptomic data from HD mouse models (Table 4). Our study has several limitations. While our epigenetic age analysis is not likely to be confounded by changes in cell composition, we cannot make the same claim about our WGCNA analysis, although our consensus analysis across three lobes mitigates this problem. Second, we studied only a relatively small number of individuals because it is very difficult to secure brain samples from human post mortem HD cases. Third, we focused on CpG methylation as opposed to hydroxy methylation (5hmC). It is noteworthy that the brain has the highest 5hmC levels in the body [56-58] and non-CpG methylation is prominent in neuronal tissue [56, 59, 60].

We can only speculate on why striatal samples do not seem to exhibit accelerated epigenetic aging. It might reflect low statistical power (due to small sample sizes), it might reflect severe neuronal loss, or it might suggest that epigenetic age acceleration can only be detected at the early stages of the disease. Future epigenetic clock analyses of the striatum and of striatal neurons should focus on the early stage of striatal degeneration (Vonsattel stage 0-1) or employ HD mouse models in which MSN cell loss is not a major feature. Studies in the rat striatum suggest that normal aging modulates the neurotoxicity of mutant huntingtin [12]. Future studies could explore whether the onset of HD can be delayed by slowing down the epigenetic aging rate. The positive youth-promoting side effects of such a treatment (delayed aging) would probably be attractive to most patients.

Overall, our study strongly suggests that HD pathogenesis is associated with large scale DNA methylation changes and with an accelerated epigenetic age in brain tissue. It remains to be seen whether epigenetic age acceleration is prognostic of age of onset or the rate of disease progression.

## METHODS

### Sample collection

Postmortem brain samples from HD and AD cases and neurologically normal controls were collected at UCLA (n=218 samples from 32 individuals) and University of Auckland (n=257 samples from 33 individuals). The UCLA samples were provided by the Brain tissue and CSF resource/bank of the Mary Easton Alzheimer Disease Research Centre at UCLA (by H. Vinters).

Cubes 3×3×3mm with approximate mass of ~30 mg were cut from histological specimens collected during necropsies. Tissue samples were frozen and stored at −80C. In order to avoid batch effects, all tissue samples were shipped to the same UCLA core facility for DNA extraction and DNA methylation profiling. The Auckland samples were obtained from the Neurological Foundation of New Zealand Human Brain Bank (University of Auckland, NZ). The tissue used for this study had been processed according to a detailed protocol, which has been previously published [61, 62], dissected into blocks, snap frozen on dry ice, and stored at −80°C.

Age of HD motoric onset was available for 21 subjects from the NZ tissue bank (median age=38, ranging from 15 to 70). A total of 475 Illumina arrays were generated from 65 individuals (26 HD, 18 Alzheimer's disease, and 21 controls). After adjusting for chronological age, we could not detect an age acceleration effect due to AD status (Supplementary Figure 4). We profiled the following brain regions: caudate nucleus (n = 29 arrays), cingulate gyrus (n=33), cerebellum (n=42), hippocampus (n=33), parietal cortex (n=64), frontal lobe (n=70), occipital cortex (n=43), temporal cortex (n=37), midbrain (n=26), motor cortex (n=33), sensory cortex (n=33), and visual cortex (n=32). We also grouped the samples into broader categories: parietal lobe (parietal lobe and sensory cortex), frontal lobe (right frontal lobe, left frontal lobe, frontal gyrus, motor cortex), occipital lobe (occipital lobe and visual cortex). In our WGCNA analysis, we focused on 3 lobes for which sufficient sample sizes (n>=75) were available: parietal (n=97), frontal (n=103), and occipital (n=75). We omitted temporal samples from the WGCNA analysis due to the relatively low sample size (n=37).

### Ethics review and IRB

All individuals whose brains reside in the UCLA tissue bank (or their legal next-of-kin) signed the “Consent for Autopsy” form by the Department of Pathology at UCLA, and research procurement was performed under IRB Research Protocol Number 11-002504. Further, the epigenetic analysis is covered by IRB Research Protocol Number: 19119.

The studies using tissue from the Neurological Foundation Human Brain Bank was approved by the University of Auckland Human Participants Ethics Committee Ref #011654. All tissue was obtained with full informed consent of the families.

### DNA extraction

AllPrep DNA/RNA/miRNA Universal Kit (Qiagen, cat # 80224) was used for the DNA extractions for frozen tissue samples. 30mg of frozen tissue was lysed with 600uL guanidine-isothiocyanate–containing Buffer RLT Plus in a 2.0mL micro centrifuge tube, and homogenized by using TissueLyser II (Qiagen) with 5mm stainless steel beads. Tissue lysate was continued with the AllPrep protocol for simultaneous extraction of genomic DNA and total RNA using RNeasy Mini spin column technology.

### DNA methylation data pre-processing

Our novel DNA methylation data have been posted on Gene Expression Omnibus (GSE72778).

Bisulfite conversion using the Zymo EZ DNA Methylation Kit (ZymoResearch, Orange, CA, USA) as well as subsequent hybridization of the HumanMethy-lation450k Bead Chip (Illumina, SanDiego, CA), and scanning (iScan, Illumina) were performed according to the manufacturers protocols by applying standard settings. DNA methylation levels (β values) were determined by calculating the ratio of intensities between methylated (signal A) and un-methylated (signal B) sites. Specifically, the β value was calculated from the intensity of the methylated (M corresponding to signal A) and un-methylated (U corresponding to signal B) sites, as the ratio of fluorescent signals β = Max(M,0)/[Max(M,0)+Max(U,0)+100]. Thus, β values range from 0 (completely un-methylated) to 1 (completely methylated) [63].

### DNA methylation age and epigenetic clock

DNA methylation levels give rise to particularly promising biomarkers of aging since chronological age (i.e. the calendar years that have passed since birth) has a profound effect on DNA methylation levels in most human tissues and cell types [13-17, 35, 64-67]. Several recent studies propose to measure accelerated aging effects using DNA methylation levels [18, 68, 69]. Here we use the epigenetic clock method (based on the DNAm levels of 353 CpGs) because a) it is largely unaffected by differences in cell composition and b) it applies to all brain regions. The method applies to two commercially standardized methylation platforms: the Illumina 450K and 27K arrays. The epigenetic clock method is an attractive biomarker of aging because (1) it applies to most human tissues; (2) its accurate measurement of chronological age is unprecedented [18]. The following results suggest that the epigenetic clock captures aspects of biological age. The epigenetic age of blood has been found to be predictive of all-cause mortality even after adjusting for a variety of known risk factors [70, 71]. Further, the blood of the offspring of Italian semi-supercentenarians (i.e. individuals who reached an age of at least 105) has a lower epigenetic age than that of age-matched controls [72]. The epigenetic age of blood relates to cognitive and physical fitness in the elderly [73] and to Parkinson's disease status [52]. The utility of the epigenetic clock method has been demonstrated in applications surrounding obesity [19], Down syndrome [51], and HIV infection [53].

Predicted age, referred to as DNAm age, correlates with chronological age in sorted cell types (CD4 T cells, monocytes, B cells, glial cells, neurons) and tissues and organs including whole blood, brain, breast, kidney, liver, lung, saliva [18].

Mathematical details and software tutorials for the epigenetic clock can be found in the Additional files of [18]. An online age calculator can be found at our webpage (https://dnamage.genetics.ucla.edu).

### Epigenome-wide association study

For the epigenome-wide association study and the subsequent network analysis we focused on those CpGs whose variance was at least 5×10^−4^ in at least one of the 3 lobes. This restriction resulted in 326777 CpGs retained for further analysis. DNA methylation data were adjusted for chronological age and sex by regressing methylation levels on age and sex and retaining the residuals. For association testing, we used the Kruskal-Wallis test because it is relatively insensitive to the distribution of the methylation levels and potential outliers. We used the “estlambda” function in the GenABEL R package to calculate the inflation factors [74].

### Meta-analysis

Our analysis methods make extensive use of meta-analysis. A simple yet powerful meta-analysis method, known as Stouffer's method, relies on combining the Z statistics from individual data sets (the 3 brain lobes). Specifically, for each CpG *i* and data set (brain lobe) *a,* one obtains a Z statistic *Z_ia_*, for example, by the inverse normal transformation of the p-value. Next, a meta-analysis *Z*_i_ statistic for each CpG is calculated as

$$Z_{i}=\frac{1}{\sqrt{N_{\text{sets}}}}\sum_{a=1}^{N_{\text{sets}}}Z_{ia}$$.

The meta-analysis statistic *Z_i_* is approximately normally distributed with mean 0 and variance 1; the corresponding p-value is then calculated using the normal distribution.

### Weighted Correlation Network Analysis

Weighted Correlation Network Analysis (WGCNA) [31, 32] uses as input a matrix of pairwise correlations between all pairs of CpGs across the measured samples in a data set. To minimize effects of possible outliers, we use the biweight midcorrelation[75] with argument maxPOutliers = 0.05. One then forms a “signed hybrid” pairwise co-methylation similarity that equals the correlation if the correlation is positive, and equals zero otherwise. Next the co-methylation similarity is raised to the power β=6 (WGCNA default) to arrive at the network adjacency. This procedure has the effect of suppressing low correlations that may be due to noise. The result is a network adjacency that is zero for negatively correlated CpGs and is positive for positively correlated CpGs. Adjacency of weakly correlated CpGs is nearly zero due to the power transformation.

### Consensus module analysis

Consensus modules are defined as sets of nodes that are highly connected in multiple networks; loosely speaking, one could identify the consensus module in individual network analyses across multiple sets, so the module can be said to arise from a consensus of multiple data sets [36].

Within WGCNA, consensus modules are identified using a consensus dissimilarity that is used as input to a clustering procedure. To describe our definition of the consensus dissimilarity, we introduce the following component-wise quantile function for a set of *k* matrices *A^(1)^, A^(2)^, …, A^(k)^*:

$${\text{Quantile}}_{q;i,j}={\text{Quantile}}_{q}\;(A_{i,j}^{\left(1\right)},\;A_{i,j}^{\left(2\right)},\ldots ,\;A_{i,j}^{\left(k\right)})$$.

Thus, each component of the quantile matrix is the given quantile (0 ≤ *q* ≤ 1) of the corresponding components in the individual input matrices. Using this notation, we define the consensus network corresponding to input networks *A^(1)^, A^(2)^, …, A^(k)^* and quantile *q* as

$${\text{Consensus}}_{q}\;(A^{\left(1\right)},\;A^{\left(2\right)},\ldots ,\;A^{\left(k\right)})={\text{Quantile}}_{q}({\text{cTOM}}^{\left(1\right)},\;{\text{cTOM}}^{\left(2\right)},\ldots ,\;{\text{cTOM}}^{\left(k\right)}),$$

where *cTOM* stands for calibrated Topological Overlap Measure (TOM). The calculation of *cTOM* starts with calculating the standard TOM [31] in each input data set (network). The calibration aims to make TOM values comparable between different networks. In this work we use as calibration the quantile normalization implemented in the R package *preprocessCore* [76]. We treat the independent components (say the lower triangle) of TOM for each input network as a vector of measurements corresponding to one “sample;” thus, quantiles of the calibrated TOM matrices in each network equal each other and equal the average of the corresponding quantiles in the original, uncalibrated TOM matrices.

Given the consensus network defined above, one defines the consensus dissimilarity *ConsDiss_ij_* as

$${\text{ConsDiss}}_{ij}=1-{\text{Consensus}}_{q}\;(A^{\left(1\right)},\;A^{\left(2\right)},\ldots ,\;A^{\left(k\right)})$$.

The consensus dissimilarity is used as input to average-linkage hierarchical clustering. Branches of the resulting dendrogram are then identified using the Dynamic Tree Cut algorithm [77]. Modules are labeled by (in principle arbitrary) numeric labels and, for easier visualization, also by colors. Not all CpGs will be assigned to modules; the label 0 and color grey are reserved for CpGs not assigned to any module.

### Consensus module eigenvectors

The module identification procedure results in modules containing CpGs with highly correlated methylation profiles. It is useful to summarize such modules using a single methylation profile per input data set. We use the module eigenvector *E*, defined as the left-singular vector of the standardized methylation matrix with the largest singular value[31]. Since consensus modules are defined across *k* independent data sets, one can form their summary profiles in each lobe. Thus, a consensus module gives rise to *k* eigenvectors, one in each input data set, that provide a summary “methylation value” for each sample in the data set. This allows one to relate consensus module eigenvectors to other information, for example to disease status or other traits, in each data set, and study similarities and differences between the input data sets in terms of the module-trait associations.

### Continuous measure of module membership

Module eigenvectors lead to a natural measure of similarity (membership) of all individual CpGs to all modules. We define a fuzzy measure of module membership of CpG *i* in module *I* as

$${\text{MM}}_{i}^{I}=\text{cor }(x_{i},\;E^{I}),$$

where *x_i_* is the methylation profile of CpG *i* and *E^I^* is the eigenvector of module *I*. This definition is applicable to every individual network (data set). The value of module membership lies between −1 and 1. Higher *MM_i_^I^* indicate that the methylation profile of CpG *i* is similar to the summary profile of module *I*. Since we use signed networks here, we consider module membership near −1 low. The advantage of using correlation to quantify module membership is that the corresponding statistical significance (p-values) can be easily computed. Genes with highest module membership are called hub CpGs. Hub CpGs are centrally located inside the module and represent the methylation profiles of the entire module.

### Module membership in consensus modules

In a consensus module analysis, we calculate the fuzzy module membership *MM* for each CpG in each data set. Thus, for each consensus analysis of 3 data sets there are 3 values for the module membership of each CpG in each module. We then use meta-analysis to summarize the 3 module memberships into a single meta-analysis Z statistic[37]. Genes with the highest module membership meta-analysis Z statistics are called consensus hub CpGs. It has been shown that consensus hub CpGs can be useful in studying functional categories associated with clinical traits[37].

### Enrichment analysis of co-methylation modules

We used Illumina-supplied probe annotation to map CpG probes to genes. Since each gene is represented by multiple CpGs (up to a thousand per gene), we applied the following stepwise procedure to represent each gene by a single CpGs.

Step 1: Apply consensus WGCNA to assign each CpG to a consensus co-methylation module. Call the consensus quantile used for this consensus analysis q. This analysis reduces the original hundreds of thousands of CpGs to typically less than 100 modules (in the brain data case, about 320k CpGs were reduced to 54 modules).

Step 2: Define an artificial module assignment where the “module” label equals the gene identifier. Thus, there is one module for each gene to which at least 1 CpG maps. Discard all CpGs that do not map to a gene.

Step 3a: For each of the artificial modules that contain at least 3 CpGs, calculate intramodular connectivity in each of the input sets. At present we don't use kME but kIM which is defined as the sum of intramodular adjacencies. This results in a vector of kIM in each input data set. Use quantile normalization to calibrate the kIM vectors across the input data sets. Then calculate the consensus of the kIM vectors using the same consensus quantile q that was used for the consensus WGCNA.

Step 3b: For each of the artificial modules that contain 2 CpGs, calculate the standard deviation of each of the CpGs in each of the input sets. This results in a vector of standard deviations for CpGs in each data set. Use quantile normalization to calibrate the std. deviation vectors across the data sets. Then calculate the consensus of the calibrated standard deviation vectors using the same consensus quantile q that was used for the consensus WGCNA.

Step 4: Represent each gene with at least 3 CpGs by the consensus hub CpG, i.e. the CpG with the highest consensus kIM. Represent each gene with 2 CpGs by the CpG with the highest consensus standard deviation. Represent each gene with 1 CpG by the single CpG. Thus, we move from 300k CpGs to about 20k representative CpGs (which are mapped in a one to one fashion to the gene identifiers).

Step 5: Assign each gene to a co-methylation module (from WGCNA) using the color label (from step 1) of the representative CpG.

Step 6: Next apply the *enrichmentAnalysis* function (from R package *anRichment*) to the genes and corresponding color labels from step 5.

We then used standard hypergeometric test (Fisher's exact test) to evaluate the significance of the overlaps of the gene-mapped methylation modules with reference gene sets including Gene Ontology, KEGG, Reactome, gene lists from [78] in the WGCNA R package, and modules from several WGCNA analyses on various HD-related gene expression data. All gene sets used in our analysis can be accessed at (https://labs.genetics.ucla.edu/horvath/htdocs/CoexpressionNetwork/GeneAnnotation/). These HD related genes as are part of the HDinHD software tool [38] (www.HDinHD.org).

Steps 3-5 are implemented in the R function “*consensusRepresentatives*” included in the package WGCNA since version 1.50. Additionally, the *anRichment* R package (https://labs.genetics.ucla.edu/ horvath/htdocs/CoexpressionNetwork/GeneAnnotation/) contains the function “*representativeCpG*” that further tailors the consensus representative selection to methylation data assayed on the Illumina Infinium 450k microarray.

## SUPPLEMENTARY DATA TABLE FIGURES AND DATASETS






