# Supplementary material for: Huntington's disease accelerates epigenetic aging of human brain and disrupts DNA methylation levels
Source: Aging (Albany NY). 2016 Jul 27;8(7):1485–504. doi: 10.18632/aging.101005 (PMC4993344; doi:10.18632/aging.101005)
Supplement: Supplementary file 1 [file aging-08-1485-s001.pdf]

SUPPLEMENTARY DATA

Supplementary Table 1. Individual level data on the subjects.

	PersonID	BrainBank	Age	Disease	PostMortemInterval	CAGlength
1	1	UCLA	65	AD	9.0	NA
2	2	UCLA	15	Control	6.0	NA
3	3	UCLA	59	AD	10.0	NA
4	4	UCLA	85	AD	22.0	NA
5	5	UCLA	100	AD	52.0	NA
6	6	UCLA	80	AD	10.0	NA
7	7	UCLA	80	AD	22.0	NA
8	8	UCLA	101	AD	28.0	NA
9	9	UCLA	64	AD	34.0	NA
10	10	UCLA	59	AD	20.0	NA
11	11	UCLA	50	HD	46.0	NA
12	12	UCLA	93	Control	22.0	NA
13	13	UCLA	85	Control	36.0	NA
14	14	UCLA	96	AD	NA	NA
15	15	UCLA	63	Control	28.0	NA
16	16	UCLA	58	HD	16.0	NA
17	17	UCLA	100	AD	26.0	NA
18	18	UCLA	81	Control	23.0	NA
19	19	UCLA	85	Control	12.0	NA
20	20	UCLA	53	Control	12.0	NA
21	21	UCLA	38	Control	24.0	NA
22	22	UCLA	62	HD	23.0	NA
23	23	UCLA	82	AD	17.0	NA
24	24	UCLA	111	AD	16.0	NA
25	25	UCLA	53	Control	23.0	NA
26	26	UCLA	64	AD	29.0	NA
27	27	UCLA	58	AD	16.0	NA
28	28	UCLA	30	HD	8.0	NA
29	29	UCLA	93	AD	4.0	NA
30	30	UCLA	65	HD	23.0	NA
31	31	UCLA	114	AD	13.0	NA

32	32	UCLA	112	AD	NA	NA
33	33	NewZealand	45	Control	12.0	NA
34	34	NewZealand	81	Control	7.0	18
35	35	NewZealand	64	Control	17.0	NA
36	36	NewZealand	36	Control	11.0	22
37	37	NewZealand	32	Control	13.0	NA
38	38	NewZealand	41	Control	16.0	NA
39	39	NewZealand	64	Control	7.0	18
40	40	NewZealand	53	Control	16.5	NA
41	41	NewZealand	73	Control	13.0	23
42	42	NewZealand	41	Control	16.0	22
43	43	NewZealand	83	Control	14.0	24
44	44	NewZealand	63	Control	16.0	NA
45	45	NewZealand	39	HD	15.0	52
46	46	NewZealand	42	HD	12.0	42
47	47	NewZealand	71	HD	16.0	42
48	48	NewZealand	53	HD	9.0	53
49	49	NewZealand	48	HD	18.0	45
50	50	NewZealand	45	HD	15.0	43
51	51	NewZealand	80	HD	9.0	40
52	52	NewZealand	43	HD	3.5	49
53	53	NewZealand	41	HD	11.0	39
54	54	NewZealand	67	HD	9.0	42
55	55	NewZealand	91	HD	18.0	40
56	56	NewZealand	62	HD	11.0	45
57	57	NewZealand	58	HD	14.0	44
58	58	NewZealand	53	HD	12.0	47
59	59	NewZealand	56	HD	16.0	46
60	60	NewZealand	51	HD	15.5	48
61	61	NewZealand	51	HD	15.0	46
62	62	NewZealand	32	HD	14.0	47
63	63	NewZealand	65	HD	14.0	43
64	64	NewZealand	62	HD	9.0	43
65	65	NewZealand	83	HD	13.0	42

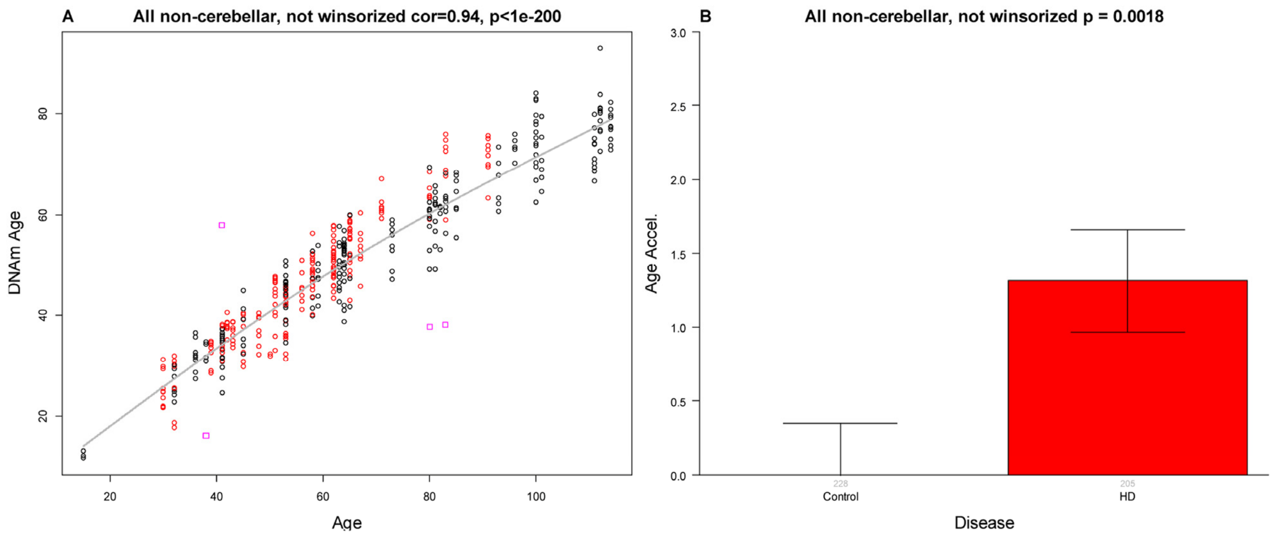

Supplementary Figure 1. Epigenetic age analysis across all non-cerebellar brain regions. (**A**) DNAm age (y-axis) versus chronological age sample collection (i.e. death). Red dots correspond to HD cases, black dots to controls, magenta dots correspond to putative outliers. The curve corresponds to a spline regression line (2 degrees of freedom) through the control samples. The scatter plot reports a Pearson correlation coefficient and corresponding p-value. Epigenetic age acceleration was defined as the vertical distance of each sample from the spline regression line. (**B**) The bar plot presents mean epigenetic age acceleration (and one standard error) versus disease status. By definition, the mean epigenetic age acceleration in controls is zero. The p-value results from a non-parametric group comparison test (Kruskal Wallis).

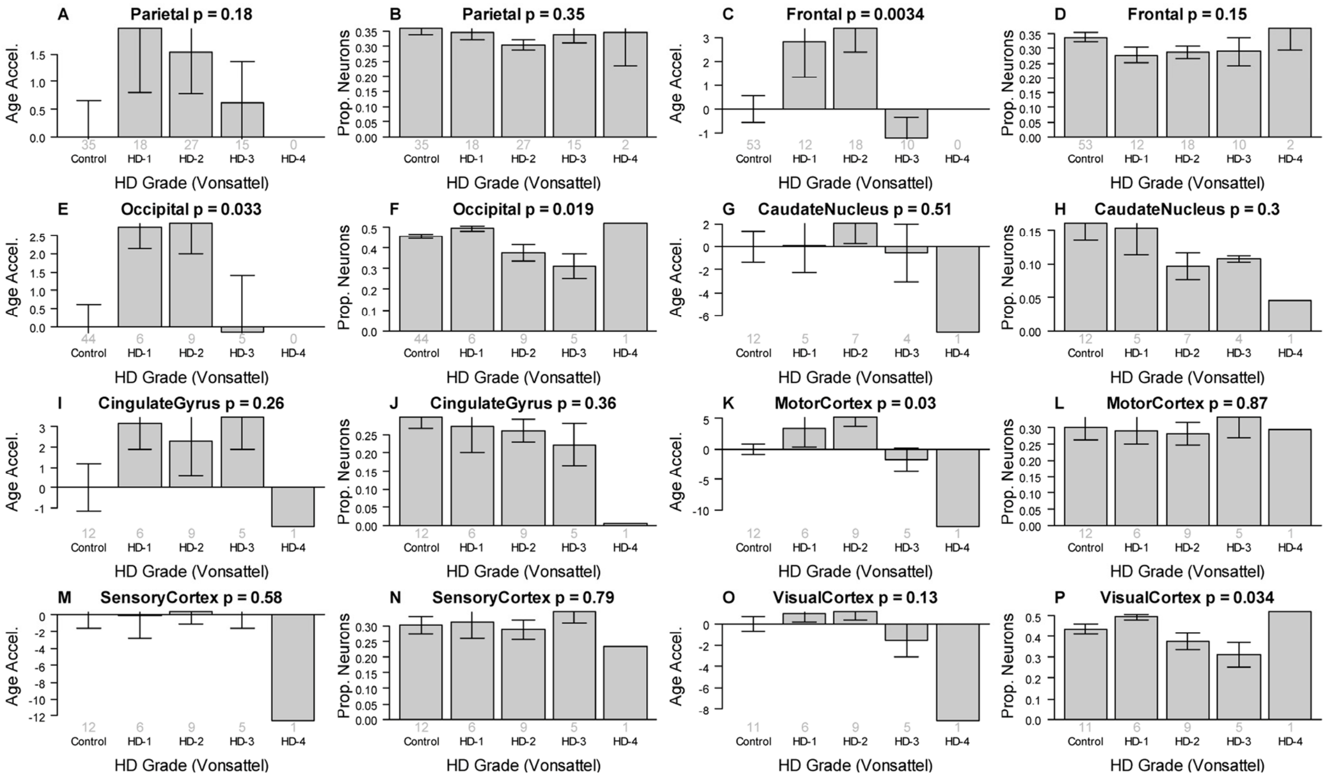

Supplementary Figure 2. HD Vonsattel grade (x-axis) versus epigenetic age acceleration and proportion of neurons. The panels correspond to different brain regions. **A,C,E,G,I,K,M,O**) Mean age acceleration (with 1 standard error) versus HD Vonsattel grade (x-axis). For the sake of comparison, the first bar reports the mean age acceleration in control samples. By definition, the mean age acceleration in control samples is zero. **B,D,F,H,J,L,N,P**) Estimated proportion of neurons versus VS grade.

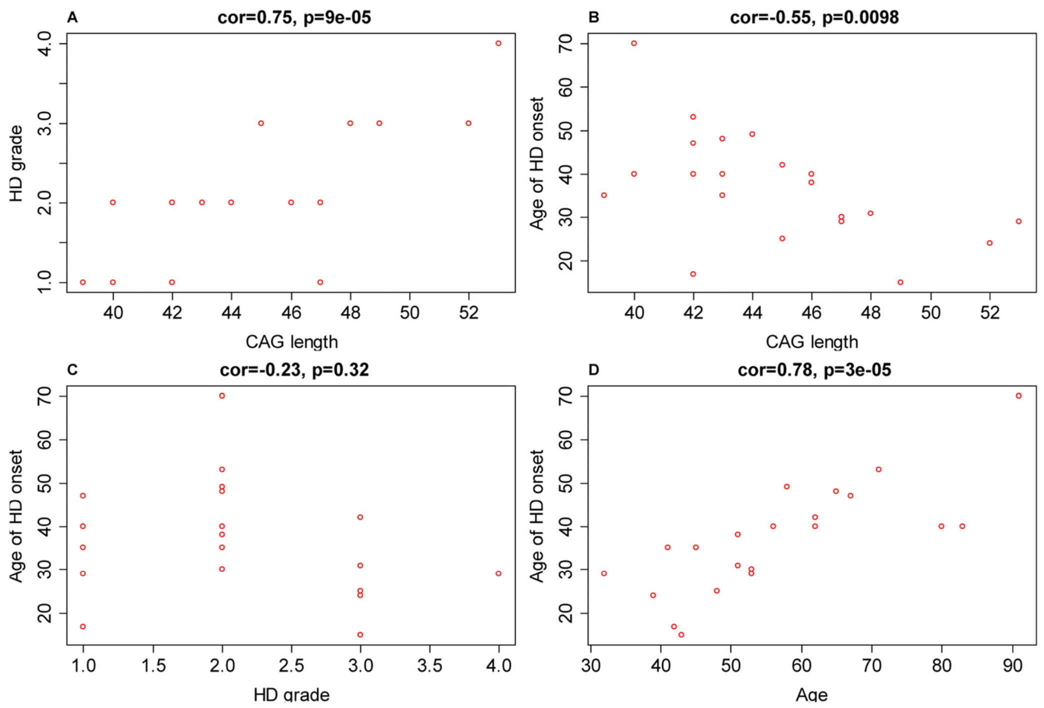

Supplementary Figure 3. Correlations between CAG length, HD grade, and age of HD onset in HD subjects. (**A-C**) Pairwise scatter plots based on 21 HD subjects from the New Zealand tissue bank. (**D**) Chronological age (at death) versus age of onset.

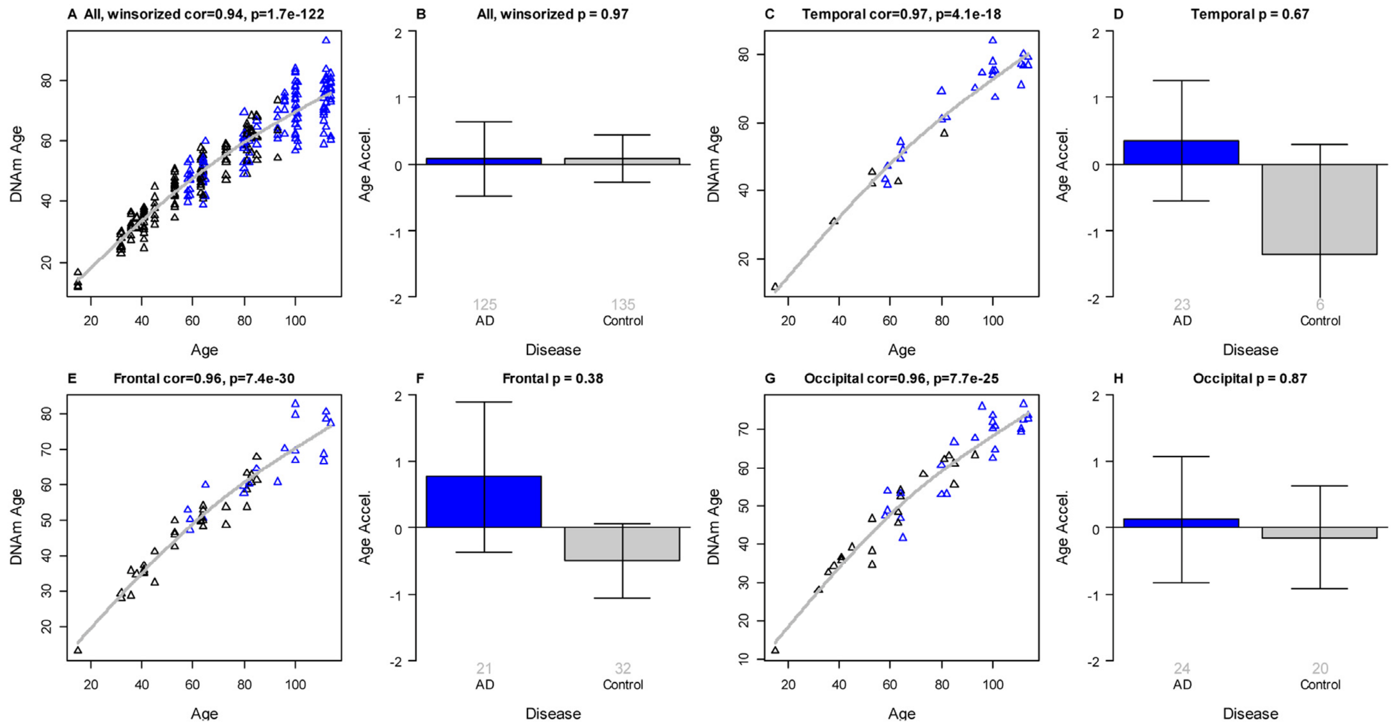

Supplementary Figure 4. Epigenetic age analysis of Alzheimer's disease. Here we removed all HD samples. Blue and red dots correspond to Alzheimer's disease and control samples respectively. The bar plots report Kruskal Wallis test p-values. Results for (**A,B**) all brain regions, (**C,D**) temporal lobe, (**E,F)** frontal lobe, (**G,H**) occipital lobe.

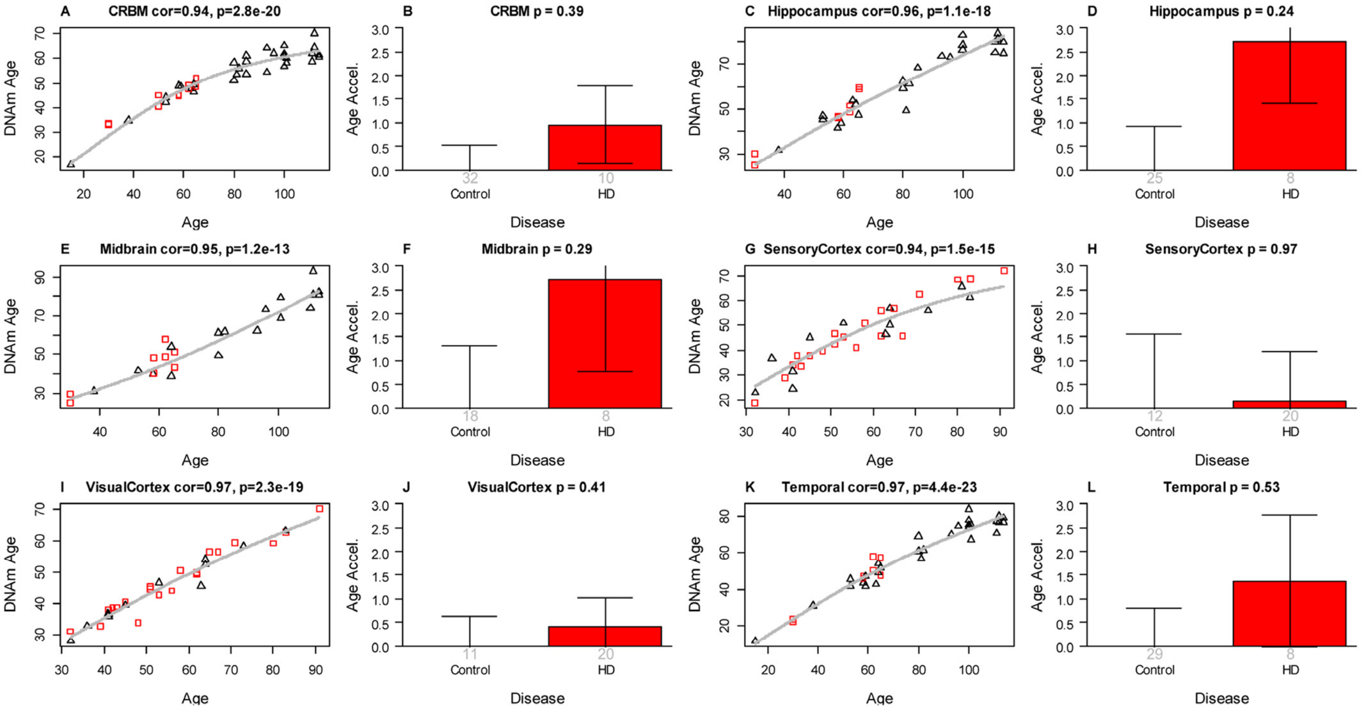

Supplementary Figure 5. Epigenetic age analysis in brain regions that lead to insignificant results. Here we used winsorized DNAm age estimates. We use ANOVA instead of the Kruskal Wallis test in the bar plots because of the low group sizes. Samples with HD grade 4 were removed from the analysis.

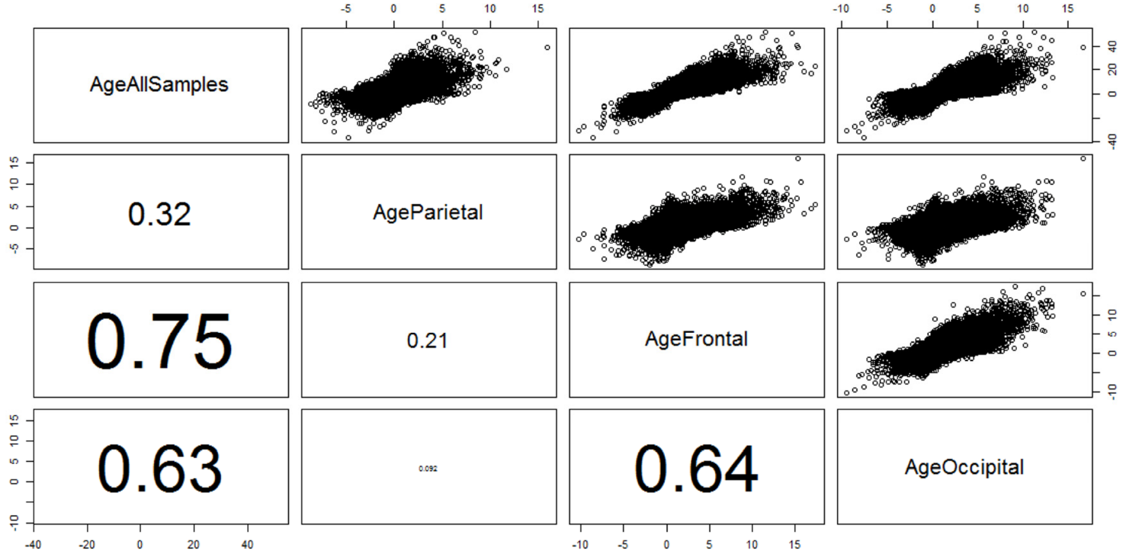

Supplementary Figure 6. The effect of chronological age on DNA methylation levels are preserved across lobes. The axis of each plot shows the signed log (base 10) transformed p-value of a correlation test. The panels above the diagonal show scatter plots. The numbers in the lower diagonal show the corresponding correlation coefficients.

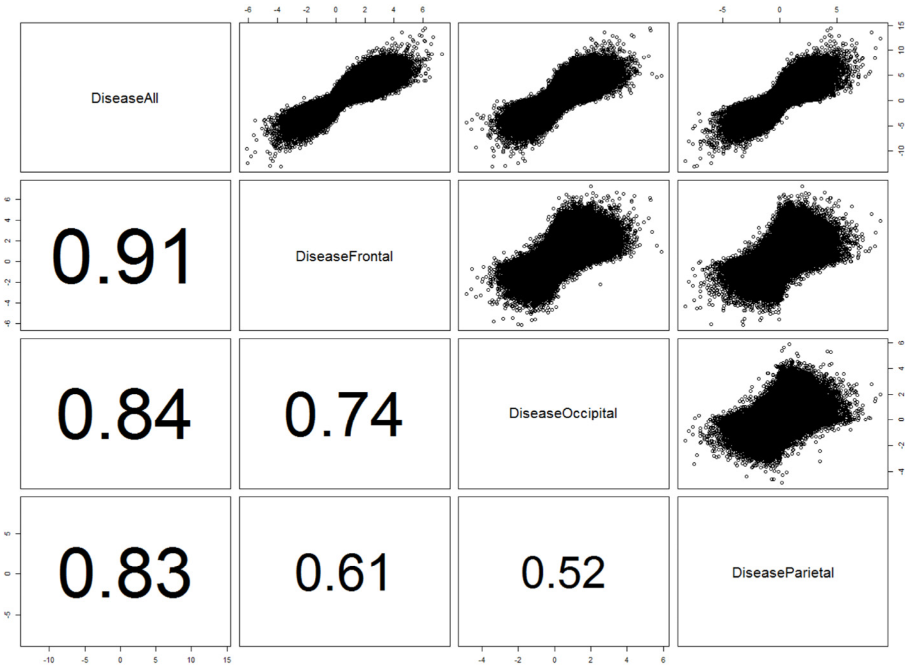

Supplementary Figure 7. The effect of HD status on DNA methylation levels are preserved across lobes. The axis of each plot shows the signed log (base 10) transformed p-value of a group comparison test. The panels above the diagonal show scatter plots. The numbers in the lower diagonal show the corresponding correlation coefficients.

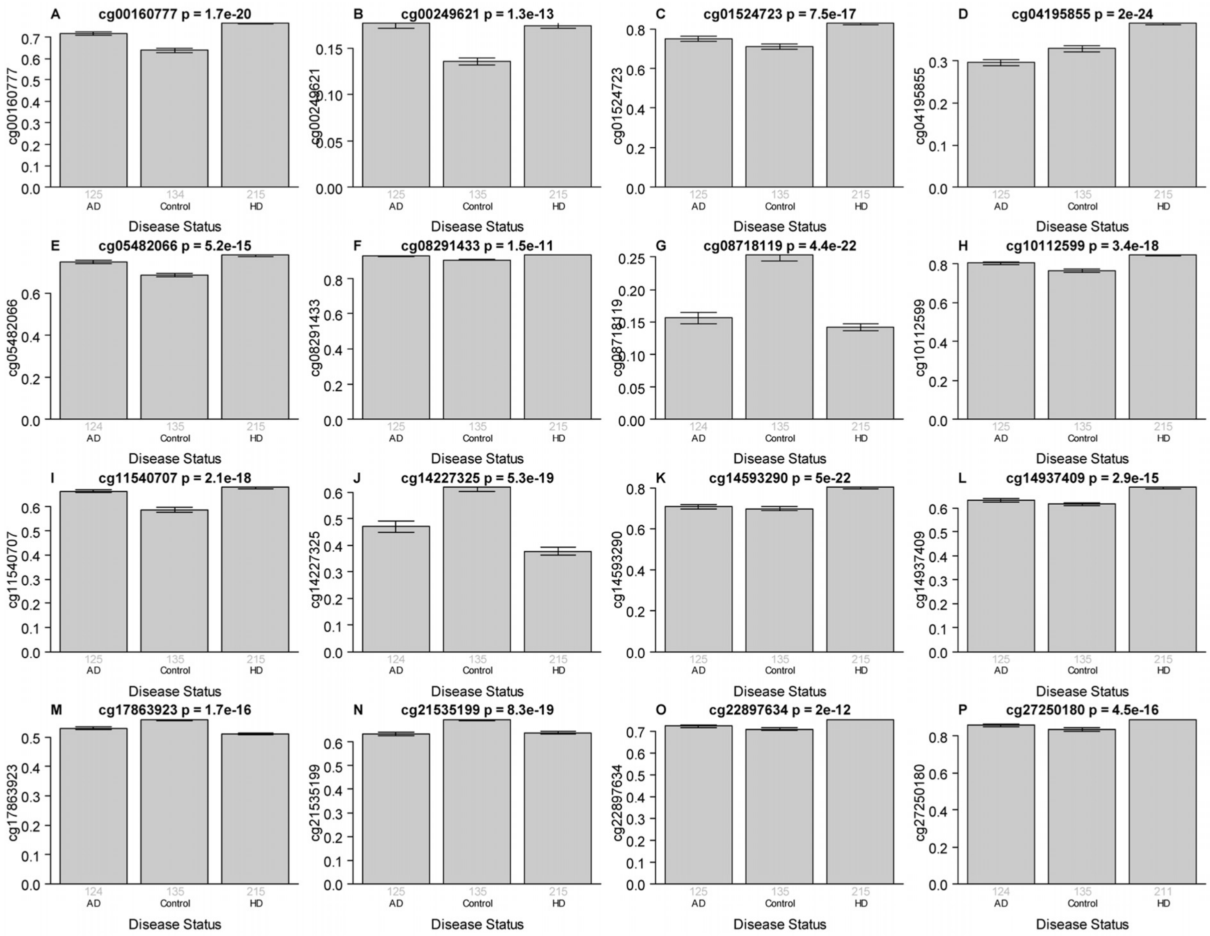

Supplementary Figure 8. The most significant CpGs resulting from our EWAS meta analysis versus disease status. The panels correspond to the CpGs in Table 3. Each bar plot relates the DNA methylation levels (beta values) to disease status. This statistical analysis (and p-values) differs from those presented in Table 3 for the following reasons. First, the y-axis shows beta values that were not adjusted for chronological age. Second, the grouping variable (x-axis) takes on 3 levels (HD, Alzheimer's disease, and controls) whereas a binary grouping variable (HD vs non-HD) was used to select the CpGs of Table 3. Brain regions are ignored (i.e. lumpted together).

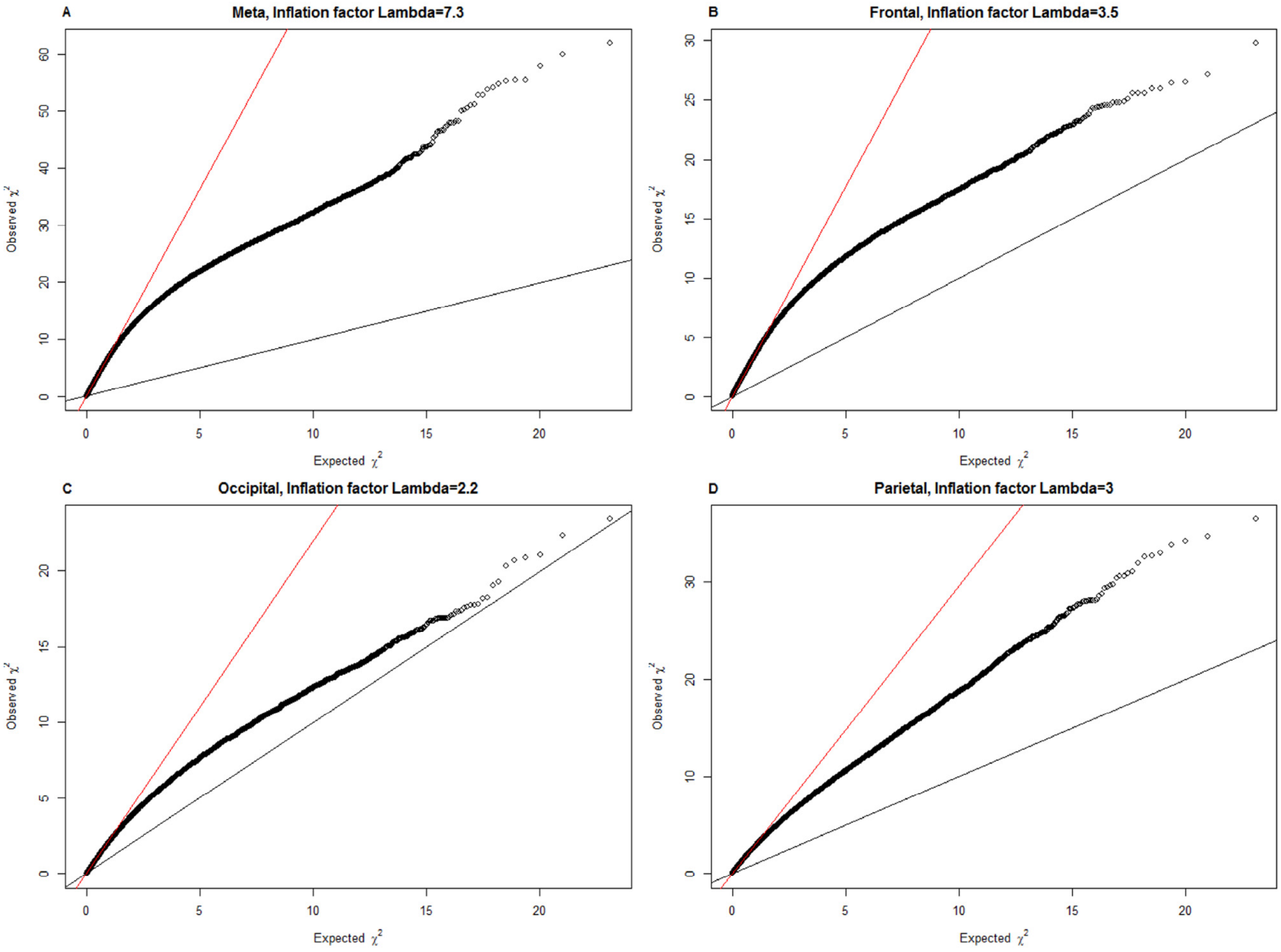

Supplementary Figure 9. Estimation of inflation factors (lambda values) for the EWAS of HD. The panels correspond to the respective studies in Figure 3. Each panel shows the estimated lambda inflation factor and plot resulting from the estlambda R function. Lambda factors for (**A**) p-values resulting from a meta analysis across 3 lobes (frontal, parietal, and occipital lobe), (**B**) frontal lobe, (**C**) occipital lobe, (**D**) parietal lobe.

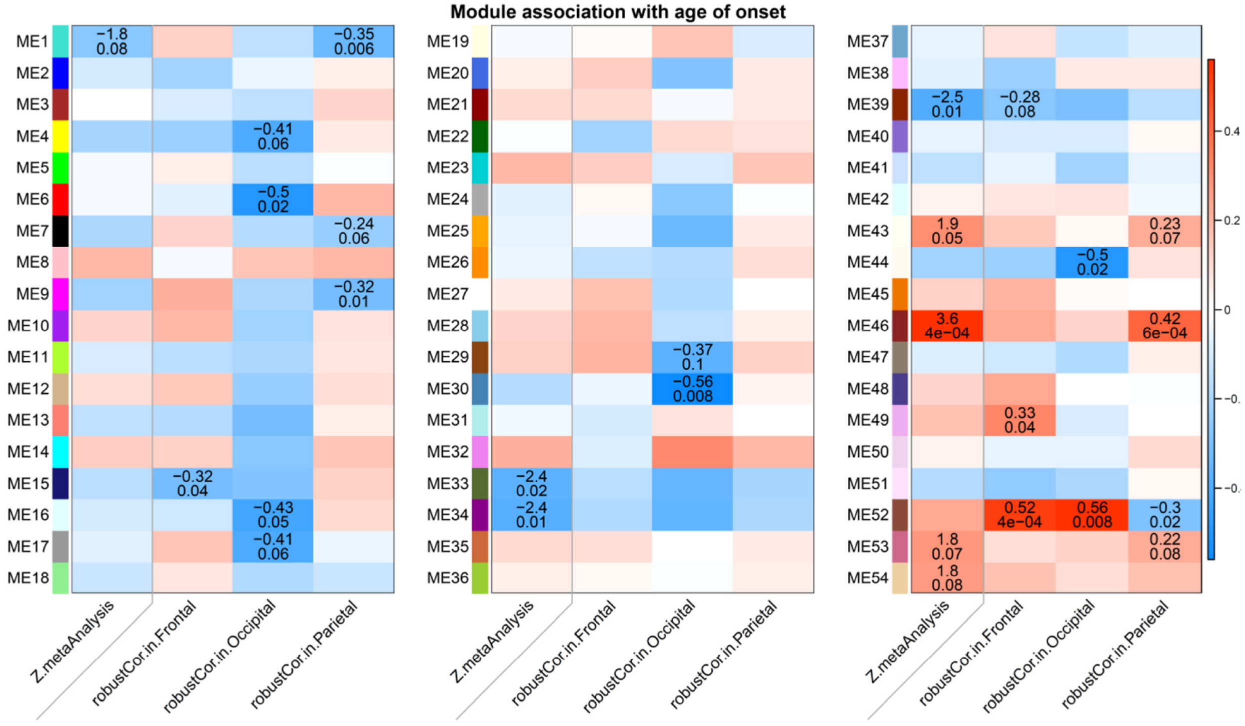

Supplementary Figure 10. Correlations between module eigengenes and age at HD motor onset. The Figure is analogous to Figure 5 but the correlation values and Z statistics refer to age of onset. The first column in each panel reports a Z statistic. The remaining columns report robust correlation coefficients in different brain regions.

Supplementary Data Sets

Please browse the full text version of this manuscript to see links to Supplementary Files:

Supplementary File 11. EWAS results for HD status in multiple brain regions. The file reports Kruskal Wallis test p-values and corresponding Z statistics for samples from the frontal lobe, occipital lobe, and parietal lobe. Further, it reports the meta analysis results across the three brain region. The meta-analysis p-valuea should be interpreted as a descriptive (hypothesis promoting) rather than inferential measure since the analysis did not adjust for the fact that multiple samples were collected from each individual. Results are reported for 326777 CpG from the Illumina Inf450k array, which satisfied our filtering criteria (high variance, few missing values).

Supplementary File 12. Module membership information for intramodular hubs in the consensus modules. The column correspond to the consensus modules presented in Figure 5. The Z statistics result from the application of the "consensusKME" R function. High positive Z statistics indicate that the CpG is an intramodular hub in the respective module. A CpG can be a hub in multiple modules. The module membership measure should be interpreted as a fuzzy measure of module membership. Due to space constraints, we only report those CpGs for which the absolute value of the Z statistic exceeds 17 for at least one of the modules.
